# Physiological Basis of Skeletal Muscle Electrical Stimulation in Heart Failure with Reduced Ejection Fraction: Established Mechanisms, Extrapolated Mechanisms and Translational Priorities

**DOI:** 10.3390/biomedicines14102310

**Published:** 2026-10-12

**Authors:** Damian Sendrowski, Dariusz Kozłowski

**Affiliations:** 1 Institute of Health Sciences, Pomeranian University in Słupsk, 76-200 Słupsk, Poland; 2 Department of Cardiology, Coronary Care Unit and Cardiac Rehabilitation, Janusz Korczak Voivodeship Specialist Hospital in Słupsk, 76-200 Słupsk, Poland; 3 Department of Cardiology and Electrotherapy, Faculty of Medicine, Medical University of Gdańsk, 80-210 Gdańsk, Poland; dkozl@gumed.edu.pl

**Keywords:** heart failure with reduced ejection fraction, neuromuscular electrical stimulation, functional electrical stimulation, skeletal myopathy, sarcopenia, ergoreflex, muscle sympathetic nerve activity, cardiac rehabilitation, whole-body electromyostimulation

## Abstract

Skeletal muscle electrical stimulation, delivered as neuromuscular electrical stimulation (NMES) or functional electrical stimulation (FES), is one of very few interventions in heart failure that produces sustained contractile work in the periphery without volitional effort and, in low-frequency configurations, without a measurable rise in cardiac output. Three decades of clinical evaluation have produced consistent signals of improved muscle strength, walking distance and quality of life. The physiological rationale that underpins these observations has advanced much less. This review separates what has been demonstrated in human skeletal muscle in heart failure with reduced ejection fraction (HFrEF) from what has been extrapolated from healthy volunteers, chronic obstructive pulmonary disease, critical illness and ageing muscle. We frame stimulation within the muscle hypothesis of chronic heart failure, in which peripheral myopathy drives ergoreflex overactivity, sympathetic excitation and exertional symptoms, and we show that the decisive mechanistic question, whether a programme of stimulation attenuates ergoreflex sensitivity, has never been tested, although two sham-controlled microneurographic studies indicate that stimulation acutely lowers muscle sympathetic nerve activity. Four further gaps are identified: a single biopsy-controlled randomised dataset in advanced heart failure, a single in vivo spectroscopic study in which muscle energetics did not change, absence of contemporary molecular phenotyping of anabolic and proteolytic signalling in stimulated HFrEF muscle, and drift of the recent evidence base towards decompensated, frail and older populations with mixed ejection fraction. Against the background of the 2026 European Society of Cardiology guidelines, which recommend exercise-based cardiac rehabilitation for all stable patients and mention electrical muscle stimulation only as an option for very weak patients, we propose a research agenda in which stimulation dose is reported as a biological variable and muscle afferent physiology becomes the primary mechanistic endpoint.

## 1. Introduction

Exercise training within multidisciplinary, exercise-based cardiac rehabilitation carries a Class I recommendation in the 2026 European Society of Cardiology (ESC) guidelines for the management of heart failure, which recommend personalised training for all stable patients without contraindications and state that cardiac rehabilitation should be considered as soon as safely possible after an episode of decompensation [1]. The first ESC guidelines devoted specifically to cardiac rehabilitation, published in the same year, recommend cardiac rehabilitation in chronic HFrEF to reduce all-cause hospital admission and to improve peak oxygen uptake and quality of life, and they recommend it in patients with frailty who have an indication for rehabilitation [2]. The most recent Cochrane synthesis of 60 trials confirms improvements in health-related quality of life and reductions in hospital admission with exercise-based rehabilitation, while noting that the evidence base is dominated by patients with reduced ejection fraction in New York Heart Association (NYHA) class II or III [3]. The largest randomised outcome trial in the field, HF-ACTION, demonstrated modest reductions in adjusted composite clinical endpoints with supervised aerobic training, alongside the practical difficulty of sustaining adherence over time [4]. The patients least able to comply are frequently those with the greatest peripheral impairment: advanced symptoms, muscle wasting and exertional breathlessness at low workloads [5].

Transcutaneous electrical stimulation of the lower limb muscles occupies an unusual position in this field. It produces contractile work in a defined muscle group without voluntary effort and without central motor command, and in the low-frequency configurations most often used in cardiology, it does so without a meaningful rise in cardiac output [6]. It is therefore not an alternative dose of the same intervention but a mechanistically distinct one: a peripheral intervention with, at most, a small central stimulus. Whether that distinction is a therapeutic advantage or a therapeutic ceiling is the central unresolved question in the field.

Four modalities recur in this literature and are distinguished throughout this review because they engage different tissues and impose different physiological loads. Neuromuscular electrical stimulation (NMES) denotes transcutaneous current above the motor threshold that evokes visible contraction of the target muscle. Functional electrical stimulation (FES) is used in the clinical heart failure literature for the same suprathreshold modality applied to the lower limbs, and the two terms are treated as equivalent here. Transcutaneous electrical nerve stimulation (TENS) operates below the motor threshold, evokes no contraction and acts only through sensory afferents. Whole-body electromyostimulation (WB-EMS) delivers suprathreshold current to multiple large muscle groups simultaneously through a wearable electrode system, typically at higher frequencies than lower-limb NMES. Findings obtained with one modality are not transferred to another in this review without explicit comment.

The guideline position on stimulation is instructive. The 2026 ESC heart failure guidelines do not mention electrical muscle stimulation at all [1]. The 2026 ESC cardiac rehabilitation guidelines address it in a single sentence of narrative text rather than in a recommendation table: in very weak patients, additional electrical muscle stimulation ‘can also be considered’ as the start of an exercise intervention, since it improves muscle strength, peak oxygen uptake, walking capacity and quality of life in advanced heart disease, with the caveat that close supervision and specific safety measures are required in patients with cardiac implantable electronic devices [2]. The 2021 position paper of the European Association of Preventive Cardiology had already stated that NMES of the lower limb muscles may be an alternative for patients with advanced HFrEF, while emphasising the heterogeneity of protocols [7]. None of these documents specifies which patients, which dose or which mechanism. That gap is the subject of this review.

Three features of the literature motivate a mechanistic review at this point. First, the clinical evidence base has grown substantially since 2016, but almost entirely in populations defined by acute decompensation, frailty or advanced age, in which ejection fraction is mixed or preserved [8,9,10,11,12]. Second, the physiological evidence base specific to HFrEF has barely grown at all. The claims routinely made about fibre-type transformation, mitochondrial biogenesis, mechanistic target of rapamycin (mTOR) signalling and satellite cell activation in stimulated heart failure muscle rest on a small number of studies, several of which were performed in other populations. Third, the 2026 ESC guidelines have redefined HFrEF as heart failure with a left ventricular ejection fraction (LVEF) below 50%, eliminating the category of mildly reduced ejection fraction [1]. Almost every stimulation trial discussed here recruited patients with an LVEF of 40% or less, and often 35% or less. Throughout this review, we therefore report the actual ejection fraction criterion of each study rather than assuming that the contemporary category is biologically homogeneous, a caution that the cardiac rehabilitation guidelines themselves raise about the rehabilitation trial literature [2].

This review complements, and does not duplicate, our recent systematic review of peripheral muscle electrostimulation in cardiac rehabilitation of older patients, which addressed clinical effectiveness, adherence and safety across cardiac diagnoses in populations with a mean age of 78 to 85 years [13]. The present article is restricted to mechanism and to HFrEF. The specific objective of this review is to set out what electrical stimulation does to the tissue it reaches, to classify each mechanistic claim about its use in HFrEF by the population in which that claim was actually demonstrated and to identify the experiments that would convert an argument by analogy into a tested physiological model. The novelty of the article lies in this explicit population-based classification and in its identification of ergoreflex attenuation as the central untested hypothesis of the field: throughout the review, attenuation of ergoreflex sensitivity by a programme of stimulation is treated as a prediction awaiting test, not as an established mechanism.

## 2. Literature Identification and Evidence Classification

This is a narrative, mechanistic review and not a systematic review or meta-analysis; no new data are presented. PubMed/MEDLINE, Scopus and the Cochrane Library were searched from inception to 15 September 2026 using combinations of the terms heart failure, cardiac failure, neuromuscular electrical stimulation, functional electrical stimulation, electrical muscle stimulation, electromyostimulation, whole-body electromyostimulation, skeletal muscle, myopathy, sarcopenia, ergoreflex, metaboreflex, mechanoreflex and muscle sympathetic nerve activity. Reference lists of retrieved reviews, meta-analyses and guidelines were examined by hand. Records were screened by title and abstract, full texts of potentially relevant publications were assessed against the scope defined above, and the final selection was agreed by both authors. Eligible clinical studies applied transcutaneous electrical stimulation in heart failure and reported physiological, functional or clinical outcomes; peer-reviewed English-language publications were considered, with one Polish-language review by our group cited for context. Physiological studies of stimulation in healthy volunteers, in chronic obstructive pulmonary disease, in critical illness and in animal models were included when they were the primary source of a claim that is repeated in the heart failure literature. The rationale for including this cross-population evidence is that the mechanistic literature specific to HFrEF is too small to be interpreted on its own. The boundary of the extrapolation is equally explicit: findings from outside HFrEF are used to characterise the stimulus and to generate hypotheses, never to support quantitative claims about patients with HFrEF, and their origin is flagged at each point of use by the classification below. Every citation was checked against the publisher, PubMed or Crossref record, and all numerical values quoted in the text were taken from the primary publication or its abstract.

Each mechanistic claim was assigned to one of three evidence tiers according to the population in which it was demonstrated, independently of the strength of the effect: Tier 1, demonstrated in patients with HFrEF, with the number of studies, their design and the actual ejection fraction criterion recorded; Tier 2, demonstrated in other human populations (healthy volunteers, older adults, chronic obstructive pulmonary disease, critical illness, cardiac surgery, heart failure with preserved ejection fraction); Tier 3, demonstrated only in animal or in vitro preparations. A claim was labelled ‘untested’ when no study in any population has measured the relevant endpoint after a programme of stimulation. This taxonomy is applied in Section 12. The tiers describe the population in which an endpoint has been measured, not the methodological quality of the studies or the strength of the evidence; a Tier 1 label may rest on a single small study, which is why the number, design and size of the contributing studies are reported alongside each claim.

Generative artificial intelligence tools were used in the preparation of graphics. Draft versions of Figures 1–4 and the graphical abstract were prepared with Perplexity Computer (Perplexity AI, Inc., San Francisco, CA, USA; 2026 release) from content supplied by the authors. All graphics were checked against the cited primary sources and revised by the authors, who take full responsibility for their content.

## 3. The Peripheral Substrate

### 3.1. Structural and Metabolic Myopathy

Skeletal muscle in chronic heart failure is not merely deconditioned, and the case that intrinsic skeletal myopathy is the principal limitation of exercise capacity has been argued from converging haemodynamic, histological and reflex evidence [14]. Muscle wasting, assessed by appendicular lean mass, was present in approximately one in five ambulatory patients with chronic heart failure in the SICA-HF cohort and was associated with reduced strength and exercise capacity independently of body mass index [15]. A meta-analysis of 11 studies and 1742 patients estimated the pooled prevalence of sarcopenia in heart failure at 34%, with a range from 10% to 69% that reflects both definitional and population differences [16]. Sarcopenia and cachexia are overlapping but non-identical phenotypes within the same population [17], and sarcopenia defined by contemporary consensus criteria [18] carries independent prognostic weight in both HFrEF and heart failure with preserved ejection fraction [19]. A low spot urinary creatinine concentration identifies a distinct muscle-wasting phenotype with a markedly different mortality risk in chronic heart failure [20], and circulating myostatin and microRNA-133a have been examined in relation to skeletal muscle measurements in patients with heart failure undergoing cardiac rehabilitation [21]. The pathophysiology has been reviewed in detail: systemic inflammation, neurohormonal activation, anabolic resistance, mitochondrial dysfunction and reduced perfusion act together, and the resulting muscle phenotype in turn worsens exercise intolerance [22,23,24]. The histological and biochemical signature of this myopathy is consistent across these reviews: a shift in myosin heavy chain composition away from slow oxidative type I fibres, loss of oxidative enzyme activity and mitochondrial density, capillary rarefaction with reduced oxygen diffusion capacity, impaired regenerative capacity and heightened fatigability of the remaining muscle [14,22,23,24]. Intramuscular fat infiltration reduces muscle quality independently of muscle mass, and the relationship between the failing heart and the wasting muscle is bidirectional, a loop summarised as the heart–muscle axis [13]. Each element of this signature is a candidate target for stimulation, and several are also candidate determinants of its effectiveness: a muscle dominated by fast, fatigable fibres responds to a given stimulation frequency differently from healthy muscle, atrophied fibres generate less evoked torque at any tolerated current, and impaired regeneration limits the hypertrophic response to any contractile stimulus.

The molecular baseline against which any stimulation effect must be judged has recently been described directly in human biopsies. In vastus lateralis samples from 20 patients with HFrEF (LVEF 35% or less), 20 patients with heart failure with preserved ejection fraction and 12 sedentary controls, proteolysis through the ubiquitin–proteasome system was increased in both heart failure phenotypes, with elevated muscle RING-finger protein 1 expression, ubiquitination and proteasome activity, the latter related to circulating interleukin-6; mitochondrial complex I activity was reduced by 53% in HFrEF, and proteolysis was most pronounced in patients with the lowest peak oxygen uptake [25]. These are the pathways that stimulation is presumed to modify, and, as Section 12 shows, none of them has yet been measured in stimulated HFrEF muscle.

### 3.2. The Relevance of the Substrate to Stimulation

The substrate matters for a specific reason. Electrical stimulation delivers a mechanical and metabolic stimulus to a muscle whose capacity to respond may itself be impaired. Anabolic resistance, mitochondrial dysfunction and capillary rarefaction are precisely the conditions under which a given contractile dose produces a smaller adaptive response. Any mechanistic model of stimulation in HFrEF must therefore address not only the stimulus but also the responsiveness of the target tissue, and this interaction has not been studied directly. It also implies that results obtained in healthy or ageing muscle, where responsiveness is preserved or only moderately reduced, cannot be transferred to HFrEF without qualification.

## 4. From Periphery to Centre: The Muscle Hypothesis and the Ergoreflex

The conceptual framework that gives peripheral stimulation its theoretical importance is the muscle hypothesis of chronic heart failure (Figure 1). Mechanically and metabolically sensitive afferents in skeletal muscle contribute to the haemodynamic, autonomic and ventilatory response to exercise; this contribution is exaggerated in chronic heart failure, and physical training attenuates it [26]. Ergoreceptor overactivity tracks clinical deterioration and correlates with impaired cardiorespiratory reflex control, plasma catecholamines and heart rate variability [27]. Methodological work established the reproducibility and the limits of the standard post-exercise regional circulatory occlusion protocols used to quantify metaboreflex and mechanoreflex sensitivity [28], and protocols combining passive limb movement with circulatory occlusion have since been proposed to separate the two components [29]. The construct has been reviewed comprehensively and revisited by its original proponents [30,31], and a meta-analysis of exercise training studies confirms improvement of autonomic indices with training in heart failure [32]. The 2026 ESC cardiac rehabilitation guidelines adopt the same framing, describing exertional dyspnoea in left ventricular dysfunction as having both central and peripheral components, and listing reduction of ergoreflex sensitivity among the mechanisms by which exercise training acts [2].

The haemodynamic cost of an overactive reflex in HFrEF has recently been visualised directly. During metaboreflex activation by post-exercise circulatory occlusion, left ventricular stroke volume index fell and end-systolic volume index rose in 13 patients with HFrEF studied with cardiac magnetic resonance, whereas neither changed in age-matched controls [33]. A sensitised muscle afferent system in HFrEF therefore does not merely raise blood pressure and ventilation; it impairs left ventricular output at the moment it is most needed.

The implication for stimulation is direct. If exertional symptoms in HFrEF are generated in substantial part by an overactive muscle reflex arising from a diseased periphery, then an intervention that repairs the periphery should reduce reflex sensitivity and therefore symptoms, without needing to alter central haemodynamics. This is the strongest available theoretical argument for NMES in HFrEF. It is also, as Section 8 sets out, untested as a training effect.

## 5. The Stimulus Itself: Biophysics, Recruitment and Dose

A recurring weakness in the cardiological literature is the treatment of ‘stimulation’ as a single intervention, such that a 4 Hz continuous protocol and a 50 Hz tetanic protocol are reported under one heading and pooled in one analysis. The physiology does not support that treatment. This section sets out what the delivered current actually does, which of the widely repeated claims about it are supported and why the parameter set, rather than the modality, determines the biological response (Figure 2).

### 5.1. What the Current Activates

Transcutaneous stimulation does not depolarise muscle fibres directly at the intensities used clinically. It depolarises the intramuscular branches of motor axons beneath or near the electrodes, and the muscle contracts as a consequence of action potentials propagating along those axons [37]. Two corollaries follow. First, the effective dose depends on the spatial relationship between the electrode and the motor points of the target muscle, which is why motor point mapping alters evoked torque for an identical current, and why an anatomical atlas of lower limb motor points was compiled specifically to guide electrode placement [44]. Second, the same surface current applied over a nerve trunk and over a muscle belly does not recruit the same population of motor units, and the balance between peripheral and central contributions to the evoked contraction differs between the two sites [45].

The current that reaches the motor axons is only part of what is delivered. Sensory axons have comparable or lower activation thresholds, so every stimulus train generates an afferent volley alongside the efferent one [40]. The functional weight of this afferent traffic is not trivial: peripheral nerve block reduces the torque produced during repetitive stimulation, indicating that a component of the evoked contraction is generated centrally, through spinal reflex pathways, rather than by direct motor axon activation alone [41]. The afferent volley also reaches the cortex, and its effect on sensorimotor cortical excitability depends on both stimulation intensity and dose [46]. A recent reappraisal of clinical NMES practice concludes that the technique does not bypass the central nervous system at all, but generates a multitude of neurally mediated responses that contribute substantially to force generation [47].

This has a specific implication for HFrEF. If NMES obligatorily generates an afferent volley from a limb whose ergoreceptors are sensitised (Section 4), then the intervention is not a purely peripheral one in the reflex sense. Acute stimulation could in principle produce afferent-mediated sympathoexcitation, which would be the opposite of the desired effect. That the two available sham-controlled microneurographic studies in heart failure (Section 8) reported reduction rather than augmentation of muscle sympathetic nerve activity is therefore a more informative finding than it is usually taken to be, and it raises a testable question: does the chronic adaptive effect of stimulation on the muscle add to, or merely repeat, its acute afferent effect, and at what dose does the balance shift?

### 5.2. Recruitment: What Is Supported and What Is Not

Three properties of stimulation-evoked recruitment are well established and physiologically consequential. Recruitment is spatially fixed, in that the same motor units are activated in every contraction of a session rather than rotating as they do in voluntary work; it is temporally synchronous, in that activated units discharge together at the stimulation frequency rather than asynchronously; and it is non-selective with respect to motor unit type [38]. Together these properties explain the disproportionate fatigability of electrically evoked contractions and the metabolic cost of a given torque output.

The claim that stimulation reverses the voluntary recruitment order, activating large fast motor units before small slow ones, is repeated routinely in the clinical literature, including in the cardiological literature. A critical appraisal of the evidence concluded that recruitment during NMES is better described as non-selective, spatially fixed and temporally synchronous than as a reversal of the voluntary order [38,39]. What intensity does determine is the depth and extent of the activated muscle volume: low intensities activate predominantly superficial regions and higher intensities recruit deeper ones [39]. For a cardiological readership, the practical consequence is that ‘quadriceps stimulation at tolerance’ in a patient with advanced HFrEF and low current tolerance may be activating a small superficial fraction of the muscle, which is a plausible and untested contributor to heterogeneity of response in this population.

The routine assertion that a given stimulation frequency selectively targets type I or type II fibres in patients should therefore be retired, or at least restated as what it is: an inference from fibre-type physiology rather than an observation in stimulated human heart failure muscle.

### 5.3. The Parameter Space

‘Low-frequency NMES’ underdetermines the dose. At minimum, the parameters listed in Table 1 independently affect the delivered stimulus, and each should be reported [37]. Evoked torque expressed as a percentage of maximal voluntary contraction (MVC) is the most informative descriptor of intensity, because it integrates amplitude, electrode configuration and the patient’s own force-generating capacity, and the effectiveness of an NMES programme is proportional to the level of evoked torque [37,48]. It has been reported in only two heart failure trials (Table 2), which is one reason that dose–response relationships in this field cannot be reconstructed retrospectively.

Table 2 assembles the stimulation parameters and populations of the principal heart failure trials as they appear in the primary publications. Where a parameter is not stated in the publication or its abstract, it is marked ‘NR’ rather than inferred. The table makes two points visible at once: the frequency range spans more than an order of magnitude (from 2 Hz to 85 Hz), and evoked torque as a fraction of MVC is reported in two studies only.

### 5.4. Frequency and Molecular Divergence

The strongest mechanistic argument for treating low-frequency and high-frequency protocols as different interventions comes from isolated rodent muscle. Stimulation at 10 Hz for 3 h, chosen to mimic endurance training, selectively activated the AMP-activated protein kinase (AMPK) and peroxisome proliferator-activated receptor gamma coactivator 1-alpha pathway, whereas repeated 3 s bursts at 100 Hz, chosen to mimic resistance training, increased myofibrillar and sarcoplasmic protein synthesis 5.3-fold and 2.7-fold and selectively activated the protein kinase B, tuberous sclerosis complex 2 and mTOR cascade [42]. The result is from isolated rat muscle rather than from human patients, and this limitation should be stated wherever it is cited. Its importance is nonetheless considerable: it provides a molecular mechanism by which two protocols delivered by the same device, differing only in frequency and duty cycle, would be expected to produce mitochondrial and hypertrophic adaptations respectively.

In human muscle, the downstream consequences of stimulation extend to regenerative capacity. In healthy older adults, a programme of NMES improved skeletal muscle regeneration through satellite cell fusion with myofibres [62], and the same group subsequently described fibre remodelling and a specific gene expression profile in stimulated muscle of healthy elderly subjects [63]. The molecular and neural adaptations to stimulation in ageing muscle have been reviewed in detail [64]. None of these observations has been reproduced in HFrEF, and the anabolic resistance characteristic of this population (Section 3.2) makes direct extrapolation unsafe. The point is not that these pathways are irrelevant to heart failure but that their invocation in reviews of NMES in HFrEF is, at present, borrowed rather than demonstrated.

### 5.5. Haemodynamic Consequences

The haemodynamic profile follows from the parameter set rather than from the label attached to the device. Stimulation of unloaded muscle groups in healthy volunteers with a device designed to mimic shivering increased oxygen demand sufficiently to constitute cardiovascular exercise [65], so ‘haemodynamic neutrality’ is a property of a protocol, not of the modality. Conversely, low-frequency stimulation of the quadriceps and calf muscles in 14 patients with a mean radionuclide LVEF of 22.3% did not increase cardiac output during a five-week programme [6]. Higher-frequency strength protocols occupy the other end of the range; the regimen of Quittan and colleagues delivered biphasic symmetric constant-voltage impulses of 0.7 ms pulse width at 50 Hz with a 2 s on and 6 s off duty cycle to the knee extensors of seven patients with a mean LVEF of 20.1% [43]. In healthy volunteers, a 20 min session of whole-body stimulation left blood pressure, heart rate, LVEF and diastolic function unchanged while lowering blood glucose and raising lactate, indicating a metabolic rather than a haemodynamic load [66].

Both profiles have legitimate clinical uses, and they are not interchangeable. In a patient with advanced HFrEF and recent decompensation, a protocol that raises oxygen demand may be exactly what is contraindicated; in a stable patient with quadriceps weakness as the limiting factor, it may be exactly what is required. The choice is a clinical decision that the current literature does not support, because the trials that would inform it have not separated the two.

### 5.6. Consequences for Evidence Synthesis

Two conclusions follow for the design and interpretation of research in this field. First, meta-analyses that pool protocols across the frequency range are not merely introducing statistical heterogeneity; they are averaging interventions with divergent molecular targets [42] and divergent haemodynamic profiles [6,65], which is a category error rather than a precision problem. Reported heterogeneity in existing syntheses [67,68,69,70] should be read in that light. Second, any future trial in HFrEF should report, as a minimum, frequency, pulse duration, amplitude, evoked torque as a percentage of MVC, duty cycle, electrode configuration relative to motor points, session duration and total sessions completed. Without that set, a positive or negative result cannot be attributed to a dose, and the field will continue to accumulate trials that cannot be compared with one another.

## 6. Adaptations Documented in Human Heart Failure Muscle

### 6.1. Muscle Biopsy Evidence

The only randomised investigation to combine chronic low-frequency stimulation with muscle biopsy in patients with heart failure was reported by Nuhr and colleagues in 2004 [34]. Thirty-two patients with a mean LVEF of 22 ± 5% and NYHA class II to IV symptoms on optimised drug therapy were randomised to stimulation of the knee extensors and hamstrings of both legs at 15 Hz, 0.5 ms pulse width, 2 s on and 4 s off, at an intensity producing 25% to 30% of maximal voluntary force, for 4 h per day, 7 days per week, for 10 weeks, or to a control condition in which current was limited to the sensory threshold without visible or palpable contraction. Peak oxygen uptake rose from 9.6 ± 3.5 to 11.6 ± 2.8 mL/kg/min in the stimulated group and fell from 10.6 ± 2.8 to 9.4 ± 3.2 mL/kg/min in controls; six-minute walking distance, work capacity and oxygen uptake at the anaerobic threshold improved with stimulation. In the biopsies, citrate synthase activity rose, glyceraldehyde-3-phosphate dehydrogenase activity fell and the myosin heavy chain isoform pattern shifted in the slow direction, with an increase in MHC I at the expense of MHC IId/x; none of these changed in controls [34]. The same group had shown the same enzymatic and isoform shifts, with a 9% increase in citrate synthase and a fall in MHC IId/x from 28% to 22%, after 10 weeks of 15 Hz stimulation in healthy volunteers [71].

Two features of this dataset deserve emphasis. It is a sham-controlled randomised trial with tissue endpoints, and it therefore constitutes direct Tier 1 evidence that stimulation can shift the oxidative and isoform phenotype of HFrEF muscle. It is also a single study of 32 patients, published more than twenty years ago, at a dose (4 h per day) that no contemporary programme delivers, and it predates the routine availability of the molecular techniques now used to characterise anabolic and proteolytic signalling. It has never been replicated. A systematic review restricted to trials of stimulation alone, examining enzyme activity, fibre-type composition and fibre size in human lower-limb muscle across populations, found the available evidence limited and heterogeneous across both low-frequency and high-frequency protocols [72].

### 6.2. Strength, Muscle Volume and Metabolic Surrogates

Early uncontrolled and small controlled studies in severe HFrEF established basic feasibility and effect direction. In seven patients with a mean LVEF of 20.1%, eight weeks of 50 Hz stimulation of the knee extensors increased isokinetic peak torque by 13%, from 101.0 ± 8.7 to 113.5 ± 7.2 Nm, and maximal isometric strength by 20%, without adverse events [43]. In fourteen patients with a mean LVEF of 22.3%, five weeks of low-frequency stimulation for 1 h per day on 5 days per week increased peak oxygen uptake from 17.2 ± 5.3 to 19.6 ± 5.9 mL/kg/min, raised the anaerobic threshold from 12.3 to 15.2 mL/kg/min, lengthened six-minute walking distance from 419 to 459 m, and increased gastrocnemius volume from 259.4 to 273.4 cm^3^ and soleus volume from 319 to 338 cm^3^; phosphorus-31 magnetic resonance spectroscopy of the gastrocnemius was not significantly modified, and the authors inferred that the functional gain reflected increased muscle mass rather than improved muscle metabolism [6]. That spectroscopic result is rarely cited and deserves to be, because it is the only direct in vivo metabolic measurement in this literature. It should, however, be read as a non-demonstration in a small, short study rather than as a refutation of a mitochondrial mechanism; it is not inconsistent with the enzymatic shifts observed by biopsy after a much larger dose [34]. Its implication nonetheless deserves emphasis: on the available in vivo evidence, the functional gains produced by stimulation in HFrEF cannot be attributed to improved oxidative muscle metabolism, and mechanistic accounts that assume such improvement run ahead of the data.

Comparative studies subsequently reported broadly equivalent functional gains for home-based stimulation and conventional cycle training in 46 ambulatory patients with NYHA class II or III heart failure, in whom six-minute walking distance improved by 40.6 m with stimulation and 44.6 m with cycling while peak oxygen uptake changed in neither group [49], and for low-frequency electromyostimulation compared with aerobic training [50,73], with electromyostimulation producing greater improvement in patients with the lowest exercise capacity [73]. Longer stimulation exposure of the major leg muscles for at least 1 h on 5 days per week for eight weeks raised peak oxygen uptake from 19.5 to 21.2 mL/kg/min, six-minute walking distance from 415 to 455 m and quadriceps strength from 378 to 405 N in a crossover study of 10 patients with stable chronic heart failure [53], and home-based low-frequency stimulation of the quadriceps and calf muscles for 1 h daily over eight weeks was compared with bicycle training in 30 patients with NYHA class II or III heart failure [51].

## 7. Vascular and Inflammatory Effects

In 24 patients with an LVEF below 40% and NYHA class II or III symptoms randomised 2:1 to six weeks of FES or control, stimulation improved brachial flow-mediated dilatation by 38.5%, six-minute walking distance by 7.5% and Minnesota Living with Heart Failure score by 18.2%, and reduced tumour necrosis factor alpha and soluble intercellular adhesion molecule 1, indicating an anti-inflammatory component to its peripheral action [52]. The equivalent endothelial effect has been demonstrated in a placebo-controlled trial of 30 patients with heart failure with preserved ejection fraction (mean LVEF 63 ± 6%), alongside improvement in walking distance, quality of life and depressive symptoms, whereas B-type natriuretic peptide did not change significantly [54], and in a placebo-controlled pilot study of 30 patients aged 70 years or older with systolic heart failure [55]. In the largest placebo-controlled trial in the field, 120 patients aged 71 ± 8 years with a mean LVEF of 28 ± 5% were randomised to six weeks of FES or placebo and followed for a median of 383 days; mortality did not differ, but heart failure hospitalisation was lower in the stimulated group, with a hazard ratio of 0.40 (95% confidence interval 0.21 to 0.78) [56]. This remains the only cardiovascular outcome signal in the field; it derives from a single-centre trial with 40 hospitalisation events and no accompanying mortality benefit, and it should be regarded as hypothesis-generating until replicated in a multicentre setting.

The vascular findings are mechanistically important because they identify an effect that cannot be explained by muscle mass alone. Whether the endothelial change is a local consequence of repeated hyperaemia in the stimulated limb, a systemic consequence of reduced inflammatory signalling, or a marker of reduced sympathetic vasoconstrictor tone has not been resolved. The same triad of endothelial, vascular and sympathetic alterations has been proposed as a coordinated therapeutic target in chronic heart failure [74], and exercise-induced changes in the circulating proteome of patients with advanced heart failure suggest that peripheral contractile work generates systemic signals that could in principle be reproduced by stimulation [75]; whether they are has not been examined.

## 8. Autonomic Effects: What Has Been Measured and What Has Not

Two studies have recorded muscle sympathetic nerve activity (MSNA) by microneurography in patients with heart failure receiving electrical stimulation, and they address different timescales (Figure 3). In the EMSICA study, 22 patients in NYHA class III with systolic heart failure were studied in two parallel randomised, double-blind, sham-controlled protocols; 11 patients (mean LVEF 30 ± 3.6%) received NMES of the quadriceps and triceps surae at 25 Hz, 200 µs pulse width, 3 s on and 3 s off, at the maximal tolerated contraction for 5 min, and 11 patients (mean LVEF 24 ± 2.8%) received TENS at 80 Hz below the motor threshold. MSNA recorded in the contralateral leg immediately after stimulation fell from 56.7 ± 3.3 to 51.6 ± 3.3 bursts/min after NMES and from 69.7 ± 3.1 to 63.5 ± 3.5 bursts/min after TENS, in each case compared with sham, without change in blood pressure, heart rate or respiratory parameters [35]. Three years later, in 30 patients hospitalised for decompensated heart failure with NYHA class IV symptoms and an LVEF of 30% or less, stimulation of the lower limbs at 10 Hz and 70 mA for 60 min per day over 8 to 10 consecutive days improved neurovascular control, assessed by microneurography and venous occlusion plethysmography, together with walking distance, quadriceps strength and quality of life, compared with a control condition delivered at less than 20 mA [36].

These two studies answer one question and sharpen another. They answer, in the negative for stable and hospitalised patients, the concern raised in Section 5.1 that the obligatory afferent volley of stimulation might acutely increase sympathetic outflow. They also show that the acute sympathoinhibitory effect is not confined to contractile stimulation: TENS, which produces no contraction, reduced MSNA to a similar degree [35]. Part of the autonomic effect of NMES is therefore afferent rather than contractile in origin. Neither study measured ergoreflex sensitivity, and neither addresses chronic resetting of the reflex; the findings are acute, and they are not presented here as evidence that a programme of stimulation resets the ergoreflex. The question that remains is whether repeated stimulation over weeks changes the sensitivity of the ergoreflex itself, which is the mechanism the muscle hypothesis predicts and the only one that would indicate repair of the peripheral substrate rather than repeated afferent conditioning.

That question has not been asked. Exercise training in HFrEF has been shown to alter muscle metaboreflex and mechanoreflex control of sympathetic outflow with parallel changes in afferent receptor expression [76], and the neurovascular and myopathic effects of training have been reviewed together [77]. No study has applied the standard post-exercise regional circulatory occlusion protocol before and after a programme of electrical stimulation in HFrEF. The central mechanistic claim of the field is therefore still an inference from two separate studies that have never been joined.

## 9. Anti-Catabolic Effects

Stimulation reduces myofibrillar proteolysis or preserves muscle mass in several hypercatabolic states. In 26 critically ill patients evaluated by ultrasound, daily stimulation of both lower limbs attenuated the fall in cross-sectional diameter of the rectus femoris and vastus intermedius [78]. In six fully sedated patients with acute critical illness, twice-daily quadriceps stimulation of one leg for 7 days prevented the fibre-type-specific loss of cross-sectional area seen in the contralateral control leg [79]. After cardiovascular surgery, daily stimulation from postoperative day 1 to 5 altered the time course of urinary 3-methylhistidine excretion, a marker of myofibrillar breakdown, which peaked earlier in stimulated patients, and knee extensor and handgrip strength on postoperative day 7 were greater in the 61 stimulated than in the 41 non-stimulated patients of this non-randomised comparison [80]. The evidence on muscle mass is not uniformly positive: in the Catastim 2 randomised trial of 54 critically ill patients after cardiothoracic surgery, bilateral quadriceps stimulation from the first postoperative day until intensive care discharge had no effect on ultrasound muscle layer thickness, although stimulated patients regained strength faster, and regression of intramuscular oedema interfered with the thickness measurement [81]. In patients hospitalised with acute heart failure, stimulation at 10% to 20% of maximal voluntary contraction from the day after admission met prespecified haemodynamic feasibility criteria in a randomised study of 73 analysed patients, supporting the safety of early use during a catabolic phase [10].

These findings are biologically relevant to HFrEF, since acute decompensation is itself a catabolic state, but with the exception of the feasibility data none was obtained in heart failure, and this distinction is frequently lost when the results are cited. The molecular mechanisms of muscle wasting in heart failure and the rationale for stimulation as a countermeasure have been set out in review form [23,82], although the pathways invoked, including mTOR complex 1 signalling, suppression of the ubiquitin–proteasome system and mitochondrial biogenesis, have not been measured in stimulated HFrEF muscle, even though their baseline abnormalities in HFrEF muscle are now well characterised [25]. These pathways are therefore presented here as hypotheses for HFrEF, not as demonstrated mechanisms of benefit.

## 10. Clinical Translation, Safety and Its Limits

Successive meta-analyses have reported improvements in peak oxygen consumption, six-minute walking distance, quality of life and peripheral muscle strength with stimulation in chronic heart failure, with effect sizes broadly comparable to conventional training [67,68,69]. The most recent synthesis of 14 randomised trials and 518 patients found that FES of the legs improved peak oxygen uptake (standardised mean difference 0.33, 95% confidence interval 0.07 to 0.59), six-minute walking distance (mean difference 48.0 m, 95% confidence interval 28.5 to 67.6 m) and Minnesota Living with Heart Failure score (mean difference −8.2 points, 95% confidence interval −12.6 to −3.8) compared with control conditions [70]. A systematic review of stimulation applied to the muscles of ambulation in chronic heart failure and chronic obstructive pulmonary disease reached similar conclusions while emphasising methodological limitations [83], and a Cochrane review in adults with advanced progressive disease found improvements in muscle function with acceptable tolerability [84]. In severe chronic obstructive pulmonary disease, a double-blind placebo-controlled trial provides the methodological benchmark this field lacks [85].

Four limits deserve emphasis. First, when stimulation is added to exercise training in patients already able to train, incremental benefit has not been demonstrated: in the multicentre CREMS-HF study of 91 patients with a mean LVEF of 30 ± 7%, 20 sessions of 10 Hz quadriceps electromyostimulation added to 20 sessions of exercise training produced a 14% rise in peak oxygen uptake compared with 15% with training alone [57]. This is consistent with the two modalities sharing a final common pathway and with stimulation functioning as a substitute for, or a bridge to, voluntary training rather than as an adjunct to it. The practical corollary is that stimulation is best positioned for the patient who cannot yet perform, or cannot tolerate, conventional exercise training, and not as a routine supplement for patients who are already training. Second, no trial has been powered for mortality or rehospitalisation, and natriuretic peptides have been measured in very few stimulation trials; where they were measured, in HFpEF [54] and in the older cardiac populations pooled in our systematic review [13], they did not change significantly. This is best read as consistent with haemodynamic neutrality rather than as evidence of efficacy or futility, and the statement that stimulation ‘consistently’ leaves natriuretic peptides unchanged is not supported by the number of measurements available. Third, the population has drifted. Contemporary trials address advanced heart failure on inotropic support [58], hospitalised patients with severe heart failure or recent transplantation, 60% of whom carried a cardiac implantable electronic device [11], acute decompensation in older patients [10,59,60], frail older adults with acute [8] or chronic [9] heart failure and frail patients after myocardial infarction, who do not have heart failure at all [86]. Mean ejection fraction in these cohorts is frequently 40% or higher. A meta-analysis of seven randomised trials in acute heart failure found a 69.9 m improvement in six-minute walking distance (95% confidence interval 32.2 to 107.7 m) and a large improvement in quality of life (standardised mean difference 1.53, 95% confidence interval 1.03 to 2.03), with preliminary evidence of greater leg strength and no change in LVEF, a pattern consistent with a peripheral rather than a central mechanism [12]. The HFrEF-specific physiology, by contrast, still rests on work published between 1998 and 2006. Mechanistic conclusions about contemporary HFrEF therefore rest on small trials conducted a generation ago under different background therapy, read alongside recent trials conducted in populations that no longer match the HFrEF phenotype, and the two bodies of evidence should not be treated as one. The drifted population also carries a set of plausible effect modifiers that the trial literature has not addressed. Disease stage and setting determine both the catabolic state of the muscle and the deliverable dose: a stable outpatient can complete weeks of training-like stimulation, whereas a hospitalised, decompensated patient receives days of treatment in a catabolic and often fluid-overloaded state. Ejection fraction defines the phenotype in which the peripheral hypothesis was developed, and its extension to preserved ejection fraction rests on one trial [54]. Frailty and advanced age lower the tolerated current, and intolerance of the sensation was the dominant reason for dropout in older decompensated patients [59], whereas home-based stimulation in stable older outpatients achieved full adherence without cardiovascular adverse events in the trials pooled in our systematic review [13]. Sarcopenia reduces the force-generating mass on which stimulation can act, and obesity increases the distance between electrode and motor axon, so that a larger share of the tolerated current is dissipated before it reaches the muscle [37,47]. Diabetic sensory neuropathy alters both the perception of the stimulus and the safety of prolonged skin contact, and renal dysfunction adds uraemic myopathy and electrolyte shifts to the substrate. None of these factors has, to our knowledge, been tested as an effect modifier in a stimulation trial in heart failure; trialists should record them, and syntheses should treat them as prespecified sources of heterogeneity. Fourth, the therapeutic context has changed. The historical trials predate contemporary foundational medical therapy and modern device therapy, and the guidelines now recommend early rehabilitation after decompensation [1,2], so that the comparator against which stimulation must be judged is no longer inactivity but structured early mobilisation of the kind tested in REHAB-HF, where a transitional rehabilitation intervention improved the Short Physical Performance Battery by 1.5 points at three months in 349 older patients hospitalised for heart failure, 97% of whom were frail or prefrail [87].

Safety data are reassuring but modality-specific. A systematic review of NMES in patients with implantable cardioverter–defibrillators found no electromagnetic interference with device function when stimulation was applied to the thighs, with interference described only in a case report involving abdominal stimulation and subpectoral devices, and recommended an individual safety check before treatment [88]. The 2026 ESC cardiac rehabilitation guidelines cite this review when requiring close supervision in device carriers [2], and the most recent randomised trial, in which 60% of participants carried a pacemaker, defibrillator or resynchronisation device, detected no interference during two weeks of stimulation with a protocol whose frequency was increased progressively from 2 Hz to 35 Hz at 300 µs and 9 to 45 mA [11]. Skin integrity, sensory neuropathy, the acute haemodynamic state and the tolerated amplitude should be documented at each session, and adverse event definitions should be prespecified. The safety question is not the same in the two settings in which stimulation is used, and the evidence for each should be kept separate. In stable outpatients, the randomised experience summarised in Table 2 reports good tolerability, although adverse events are sparsely and unevenly reported, and the practical concerns are skin irritation under the electrodes, sensory intolerance and adherence. Electrically evoked contractions can themselves produce muscle damage, with creatine kinase rises, strength loss and soreness of a magnitude comparable to maximal voluntary eccentric exercise, a consequence of the same non-selective, synchronous and spatially fixed recruitment described in Section 5.2; the damage attenuates markedly when bouts are repeated, so it should not preclude therapeutic use, but it is a further reason to introduce intensity progressively and to monitor muscle biomarkers in frail patients [89]. In hospitalised or recently decompensated patients, stimulation is applied to immobilised patients with unstable volume status, and the feasibility criteria applied in acute heart failure were explicitly haemodynamic [10]. On physiological grounds, prudent contraindications in this setting include suspected or unexcluded deep vein thrombosis in the limb to be stimulated, uncorrected major electrolyte disturbances, which alter membrane excitability and the arrhythmic threshold, overt haemodynamic instability beyond that accepted in the feasibility studies, unstable arrhythmias and broken or infected skin at the electrode sites; cardiac implantable electronic devices require the individual safety check described above rather than blanket exclusion [88]. A workable monitoring template for the highest-risk patients exists: in our WB-EMS case in HFrEF, every session was preceded and followed by measurement of creatine kinase, high-sensitivity troponin I, N-terminal pro-B-type natriuretic peptide, C-reactive protein and venous lactate, with prespecified haemodynamic, arrhythmic and symptom-based stopping criteria that were never triggered, and all values remained within predefined safety limits across ten sessions [61]. The dominant practical limitation reported in older decompensated patients is intolerance of the sensation, which accounted for most dropouts in a real-world randomised study [59].

The alignment between the field’s recent pivot to in-hospital use and the guideline recommendation to begin rehabilitation as soon as safely possible after decompensation [1,2] has not been articulated, but it is the most plausible route by which stimulation could enter routine care: as the first contractile stimulus for the patient who cannot yet stand on a cycle ergometer, with voluntary training taking over as capacity returns. The home-based delivery demonstrated two decades ago [49,51] and again recently [9], with the high adherence recorded in older populations [13], fits the home-based and telerehabilitation delivery models that the cardiac rehabilitation guidelines now describe [2].

## 11. Emerging Modalities: Whole-Body Electromyostimulation

WB-EMS delivers current simultaneously to multiple large muscle groups through a wearable electrode vest and belts, commonly at a frequency of 85 Hz with a pulse width of about 350 µs and an intermittent duty cycle, as in the protocol described below, and has been evaluated mainly in non-athletic adults for body composition, strength and low back pain, with a systematic review of 23 articles from 14 research projects concluding that WB-EMS improves muscle mass and function while reducing fat mass and low back pain [90]. Its acute haemodynamic footprint in healthy volunteers is small, as set out in Section 5.5 [66].

WB-EMS has not been evaluated in a controlled trial in heart failure. We recently reported the first documented use of WB-EMS in HFrEF, in a 73-year-old man with recurrent non-ST-elevation myocardial infarction, an LVEF of 20% to 25% and NYHA class III symptoms, in whom ten 20 min sessions at 85 Hz, 350 µs, 4 s on and 4 s off were added to a 24-day inpatient rehabilitation programme; serial creatine kinase, high-sensitivity troponin I, N-terminal pro-B-type natriuretic peptide, C-reactive protein and venous lactate were measured before and after every session and remained within acceptable limits, and functional and echocardiographic indices improved, although concurrent interventions preclude causal attribution to WB-EMS [61]. A three-arm, sham-controlled, non-randomised trial with sequential cohort allocation (EMS-PL HF STUDY; DRKS00040045) is now recruiting 45 patients aged 60 years or older with reduced or mildly reduced ejection fraction to compare standard cardiac rehabilitation plus active WB-EMS, plus sham WB-EMS, or alone, with change in peak oxygen uptake as the primary outcome; the protocol has been posted as a preprint, and no outcome data are available [91]. In the terms of this review, WB-EMS is a high-frequency, multi-segment protocol whose dose in terms of evoked force is not yet quantifiable, and its inclusion here is as an illustration of the breadth of the parameter space, not as evidence of efficacy in HFrEF. The rationale for stimulation in the frail patient with heart failure, and the safety considerations specific to that group, were outlined in an earlier Polish-language review by our group [92].

## 12. What Is Established and What Is Extrapolated

Table 3 and Figure 4 apply the three-tier taxonomy of Section 2 to each mechanistic claim made for stimulation in HFrEF. The pattern is consistent. Functional effects (strength, walking distance, peak oxygen uptake) are Tier 1, demonstrated repeatedly in patients with an LVEF of 40% or less, although in small trials. Endothelial and anti-inflammatory effects are Tier 1 on the basis of one randomised trial in HFrEF [52], supported by trials in HFpEF and older patients [54,55]. The shift in oxidative enzyme activity and myosin heavy chain isoforms is Tier 1 on the basis of one sham-controlled, randomised trial of 32 patients [34], unreplicated for two decades. Acute reduction of muscle sympathetic nerve activity is Tier 1 on the basis of two small sham- or low-intensity-controlled studies over minutes to days [35,36]. Everything concerning anabolic and proteolytic signalling, mitochondrial biogenesis, satellite cell activation and fibre-type selectivity of frequency is Tier 2 or Tier 3, borrowed from healthy or ageing human muscle [62,63,64], from critical illness [78,79] or from isolated rodent muscle [42]. The attenuation of ergoreflex sensitivity by a programme of stimulation, which is the prediction of the muscle hypothesis and the strongest theoretical argument for the intervention, is untested in any population. The clinical outcome signal rests on one trial [56].

We wish to be precise about what this classification does and does not mean. Tier 1 status does not make a finding established in the sense of being replicated and generalisable; it means only that the observation was made in the target population. Conversely, Tier 2 and Tier 3 findings are not irrelevant; they are the hypotheses that a properly designed HFrEF study should test. The purpose of the taxonomy is to make the origin of each claim auditable, so that a reader of a future review or trial report can see at once whether a mechanism has been demonstrated in the patients under discussion or inferred from elsewhere.

## 13. Research Priorities

The gaps identified above translate into a small number of experiments, most of which are feasible within a single-centre programme.

Ergoreflex phenotyping: A trial of low-frequency stimulation in stable HFrEF with metaboreflex and mechanoreflex sensitivity as the primary mechanistic endpoint, using the established post-exercise regional circulatory occlusion protocols [26,28] or their passive-movement variants [29], would test the central hypothesis directly. A sham arm matched for sensation without contraction, as used in EMSICA [35] and by Nuhr and colleagues [34], is essential, because the acute afferent effect on sympathetic outflow is itself sensory in part.

Contemporary tissue phenotyping: Paired vastus lateralis biopsy before and after a defined stimulation dose in HFrEF, with myosin heavy chain isoform quantification, oxidative enzyme and mitochondrial complex activity, and assessment of anabolic and ubiquitin–proteasome signalling using the panel already applied to unstimulated HFrEF muscle [25], would replace a single twenty-year-old dataset [34] and would show whether the pathways borrowed from ageing muscle [62,63] operate in the presence of anabolic resistance.

In vivo metabolic measurement: Repetition of phosphorus-31 spectroscopy or near-infrared spectroscopy of mitochondrial function, given that the only direct measurement available was a non-demonstration in 14 patients over five weeks [6].

Dose reporting and protocol stratification: Every trial should report frequency, pulse duration, amplitude, evoked torque as a percentage of MVC, duty cycle, electrode position relative to motor points, session duration and sessions completed, and syntheses should separate low-frequency endurance-surrogate protocols from high-frequency strength protocols rather than pooling them [6,42,43,65]. The requirement is reinforced by the contemporary electrophysiological literature, which concludes that the forces and the adaptations evoked by the applied current vary with the stimulus parameters [93]. Table 2 shows how far current reporting is from that standard.

Restoration of HFrEF focus and modern context: Prospective recruitment by ejection fraction, with the actual criterion stated and with foundational medical therapy and device status recorded, so that mechanistic conclusions about HFrEF are not drawn from cohorts with a mean ejection fraction above 40% and so that stimulation is compared against structured early mobilisation rather than inactivity [2,87].

An integrated design: The most informative single study would combine these elements: stable, optimally treated patients with HFrEF; randomisation to standardised low-frequency stimulation with evoked torque recorded at every session or to sensation-matched sham; and, at baseline and after 8 to 12 weeks, cardiopulmonary exercise testing with the ventilatory efficiency slope, six-minute walking distance, MSNA at rest and during post-exercise circulatory occlusion, endothelial function, in vivo muscle energetics and a biopsy substudy. Ongoing trials of stimulation added to rehabilitation, including our own sham-controlled study of WB-EMS [91], could incorporate ergoreflex assessment as a secondary endpoint at modest cost, and doing so would begin to close the gap this review describes.

## 14. Conclusions

Electrical stimulation of skeletal muscle is one of the very few means of delivering contractile work to the periphery in HFrEF while leaving central haemodynamics largely undisturbed, and this makes it the natural experimental tool for testing the muscle hypothesis of chronic heart failure. The field has instead developed as a pragmatic search for an exercise substitute, and its mechanistic foundation in HFrEF has not been renewed since the middle of the first decade of this century. The 2026 ESC guidelines recommend exercise-based rehabilitation for all stable patients and early rehabilitation after decompensation, and they allow electrical stimulation for the very weak patient without specifying dose, population or mechanism. The two sham-controlled microneurographic studies available show that stimulation acutely lowers sympathetic outflow, partly through its sensory component; whether a programme of stimulation resets the ergoreflex, and whether it does so by repairing the muscle, has never been measured. The most consequential study that could be performed is also among the simplest: measure the ergoreflex before and after stimulation, and report the dose. Until that is done, the physiological basis of an intervention now written into rehabilitation guidelines remains an argument by analogy.

## Figures and Tables

**Figure 1:**
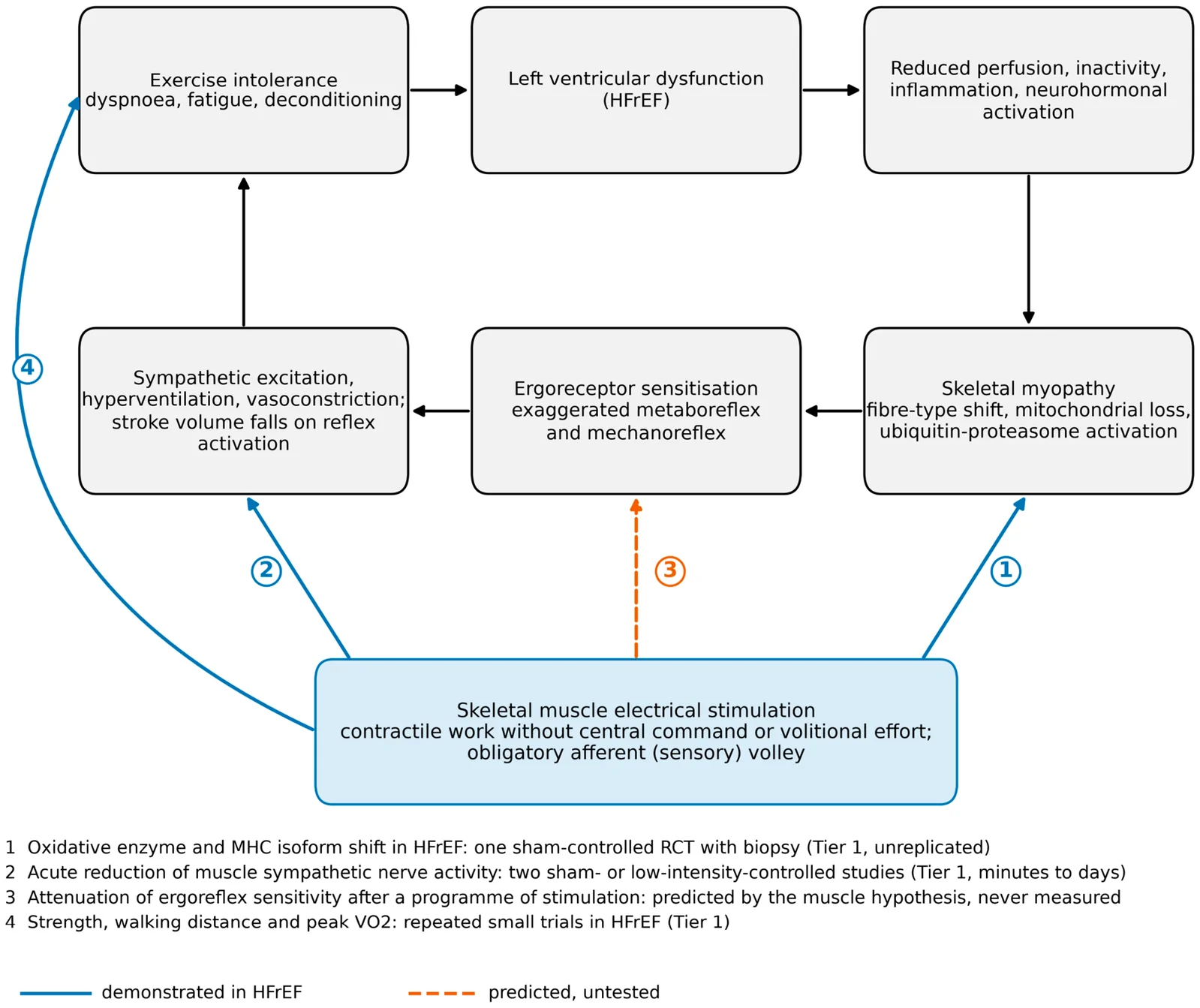
The muscle hypothesis of chronic heart failure and the points at which skeletal muscle electrical stimulation enters the loop. Solid blue arrows mark effects demonstrated in patients with HFrEF; the dashed arrow marks the effect predicted by the hypothesis but never measured. Numbers refer to the evidence summarised beneath the diagram and in Section 12. HFrEF, heart failure with reduced ejection fraction; MHC, myosin heavy chain; RCT, randomised controlled trial; VO2, oxygen uptake. Original figure prepared by the authors based on [26,27,30,33,34,35,36].

**Figure 2:**
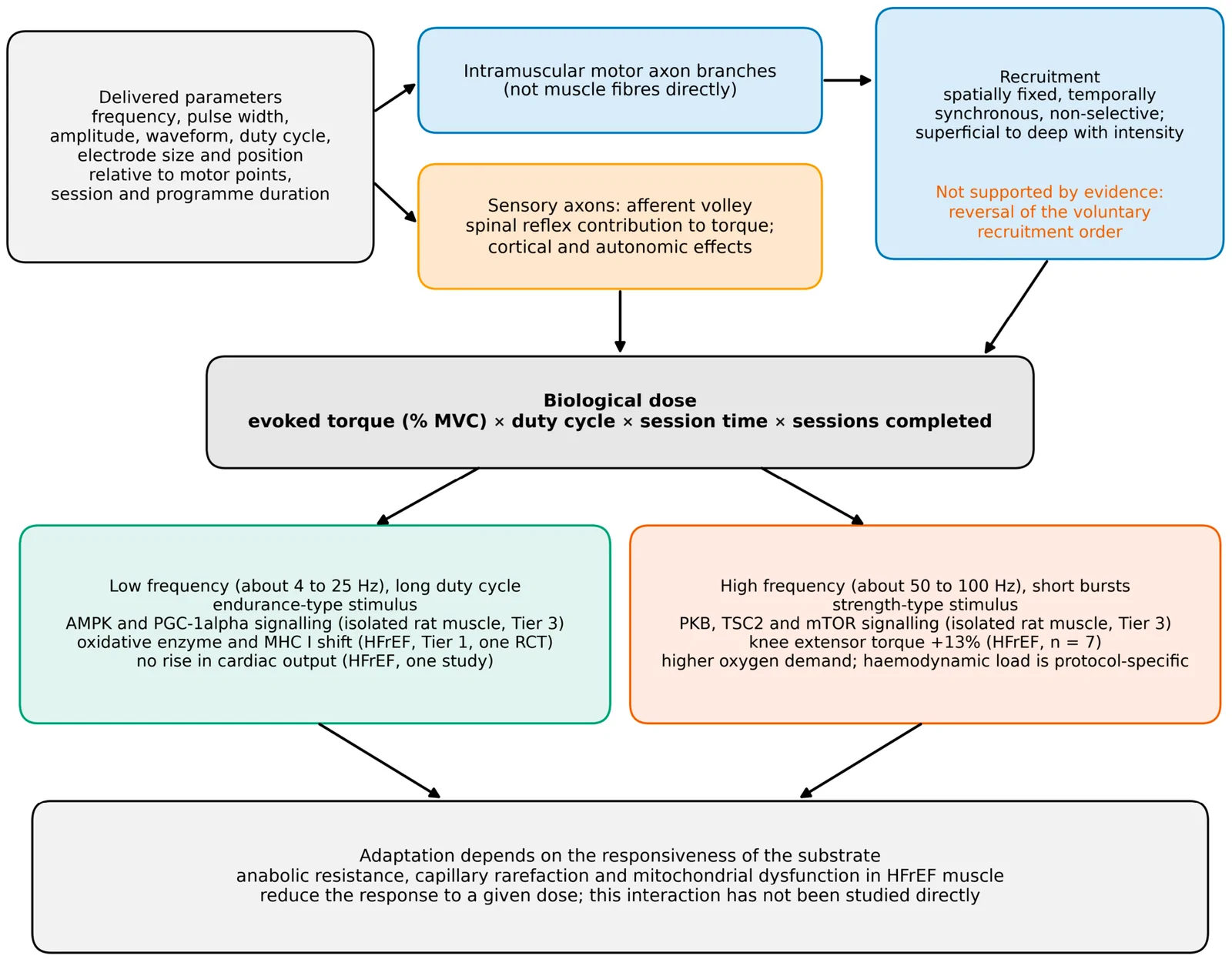
What transcutaneous electrical stimulation delivers and how the parameter set becomes a biological dose. The current depolarises intramuscular motor axon branches and, unavoidably, sensory axons; recruitment is spatially fixed, temporally synchronous and non-selective, and the claim of a reversed recruitment order is not supported. Evoked torque integrated over the duty cycle and the sessions completed is the dose to which adaptation should be related; low- and high-frequency protocols engage divergent signalling in preclinical models and carry different haemodynamic loads. Evidence tiers follow Section 2. AMPK, AMP-activated protein kinase; HFrEF, heart failure with reduced ejection fraction; MHC, myosin heavy chain; mTOR, mechanistic target of rapamycin; MVC, maximal voluntary contraction; PGC-1alpha, peroxisome proliferator-activated receptor gamma coactivator 1-alpha; PKB, protein kinase B; RCT, randomised controlled trial; TSC2, tuberous sclerosis complex 2. Original figure based on [6,34,37,38,39,40,41,42,43].

**Figure 3:**
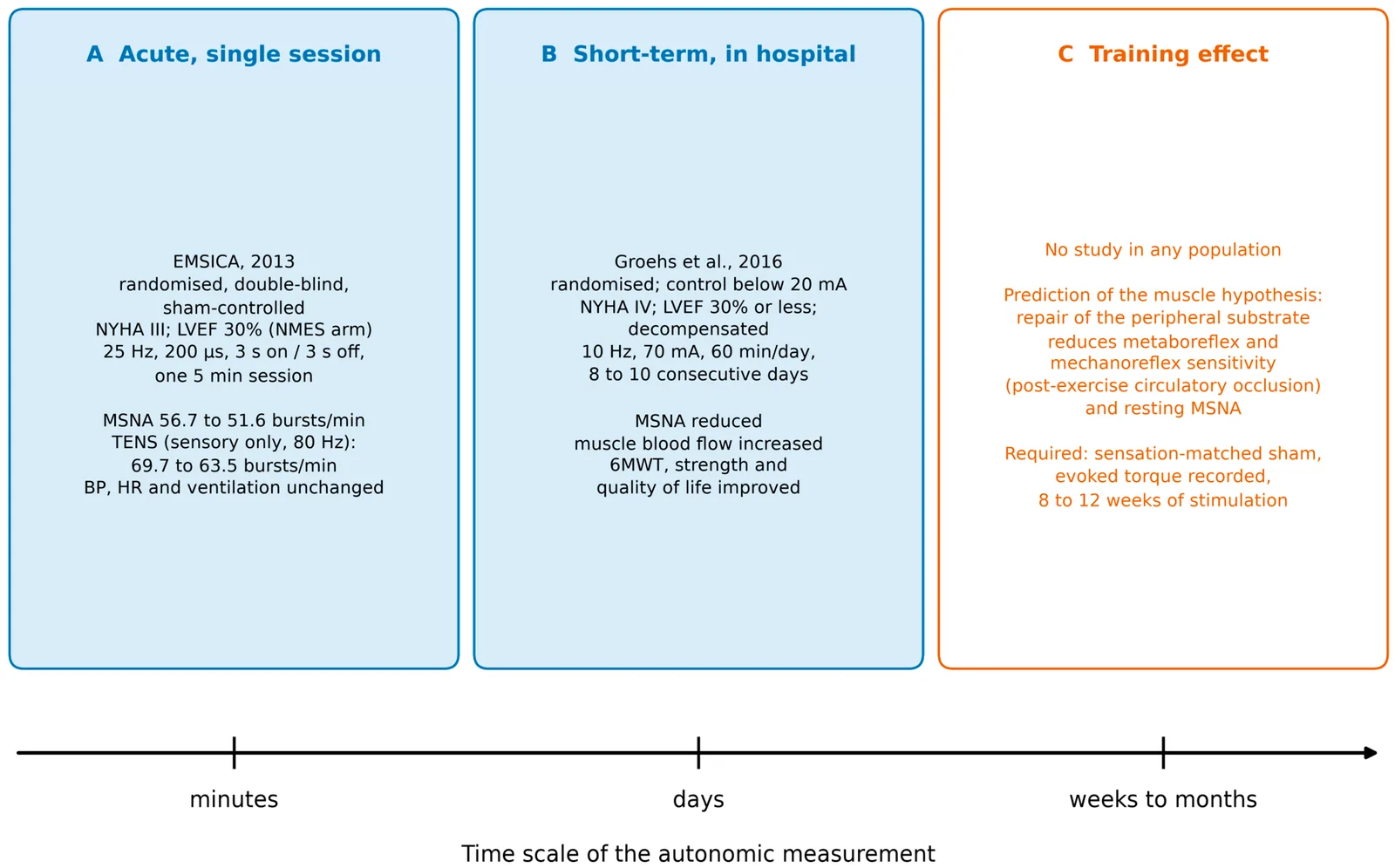
Sympathetic outflow after skeletal muscle electrical stimulation in heart failure: what has been measured at each time scale. (**A**) Acute, sham-controlled microneurography after a single 5 min session (EMSICA) [35]. (**B**) Microneurography after 8 to 10 days of daily stimulation in hospitalised patients with decompensated heart failure [36]. (**C**) No study has measured ergoreflex sensitivity or resting muscle sympathetic nerve activity before and after a multi-week programme of stimulation in any population. 6MWT, six-minute walk test; BP, blood pressure; HR, heart rate; LVEF, left ventricular ejection fraction; MSNA, muscle sympathetic nerve activity; NMES, neuromuscular electrical stimulation; NYHA, New York Heart Association; TENS, transcutaneous electrical nerve stimulation.

**Figure 4:**
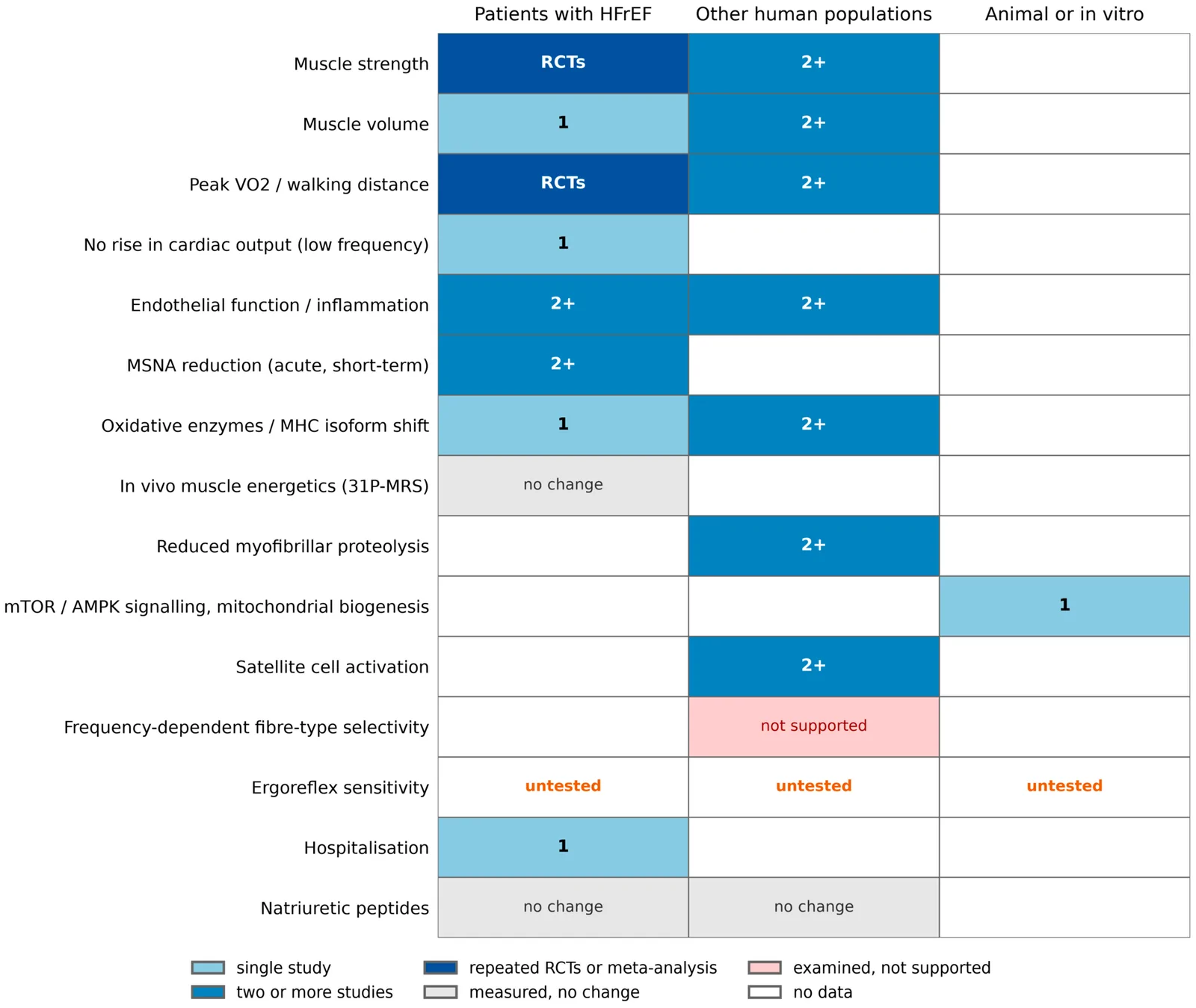
Evidence-tier matrix for the mechanistic claims made for skeletal muscle electrical stimulation in HFrEF. Rows are claims; columns are the populations in which the relevant endpoint has been measured after stimulation. Cell shading and labels indicate the number and design of the available studies, not the size of the effect. ‘Measured, no change’ denotes a non-demonstration; ‘examined, not supported’ denotes a claim that a critical appraisal of the evidence has rejected. The corresponding citations are given in Table 3. 31P-MRS, phosphorus-31 magnetic resonance spectroscopy; AMPK, AMP-activated protein kinase; HFrEF, heart failure with reduced ejection fraction; MHC, myosin heavy chain; MSNA, muscle sympathetic nerve activity; mTOR, mechanistic target of rapamycin; RCT, randomised controlled trial; VO2, oxygen uptake.

**Table 1:** Stimulation parameters that independently determine the delivered dose and should be reported in every trial. Based on physiological and methodological considerations set out by Maffiuletti [37] and Maffiuletti et al. [47].

Parameter	Physiological Role
Pulse frequency	Determines whether the contraction is twitch-like or fused (tetanic) and governs metabolic cost and fatigue for a given force; drives divergent molecular signalling in preclinical models
Pulse duration (width)	Affects the axonal recruitment threshold and the relative activation of motor and sensory axons; wider pulses recruit a larger reflex (central) contribution to torque
Current amplitude	Determines the volume and depth of activated muscle; principal determinant of evoked torque as a fraction of maximal voluntary contraction
Evoked torque (% MVC)	Integrates amplitude, electrode configuration and the patient’s own force-generating capacity; the most informative single descriptor of intensity
Duty cycle (on:off ratio)	Governs cumulative contractile work per session and the degree of fatigue accumulation
Electrode size and position	Determine current density and proximity to motor points, and hence torque for a given amplitude
Waveform and polarity	Affect comfort and therefore attainable amplitude, which is often the binding constraint in advanced heart failure
Session duration and weekly frequency	Determine total contractile work, the variable most plausibly related to adaptation
Programme duration and sessions completed	Define cumulative exposure; adherence data convert a prescribed dose into a delivered dose

**Table 2:** Populations and stimulation parameters of the principal clinical studies of skeletal muscle electrical stimulation in heart failure, as stated in the primary publications. NR, not reported in the publication or its abstract. Frequency is the pulse frequency; duty cycle is stimulation on/off time. LVEF, left ventricular ejection fraction; MVC, maximal voluntary contraction; NYHA, New York Heart Association; RCT, randomised controlled trial; TENS, transcutaneous electrical nerve stimulation; WB-EMS, whole-body electromyostimulation.

Study	Population (*n*; LVEF; NYHA; Setting)	Design and Comparator	Muscles	Frequency	Pulse Width	Intensity Criterion	Duty Cycle	Session, Frequency, Duration	Principal Finding
Maillefert et al. 1998 [6]	14; mean LVEF 22.3%; stable chronic HF	Uncontrolled before and after	Quadriceps and calf	Low frequency (Hz NR)	NR	NR	NR	1 h/day, 5 days/week, 5 weeks	Peak VO2 17.2 to 19.6 mL/kg/min; calf muscle volume increased; 31P-MRS unchanged; no rise in cardiac output
Quittan et al. 1999 [43]	7; mean LVEF 20.1%; stable chronic HF	Uncontrolled before and after	Knee extensors	50 Hz	0.7 ms	Constant voltage; NR as % MVC	2 s on, 6 s off	8 weeks (sessions NR)	Isokinetic peak torque +13%; isometric strength +20%; no adverse events
Harris et al. 2003 [49]	46; LVEF NR; NYHA II/III; stable, home-based	RCT versus home bicycle training	Quadriceps and gastrocnemius	NR	NR	NR	NR	6 weeks	6MWT +40.6 m (stimulation) versus +44.6 m (bicycle); peak VO2 unchanged in both
Nuhr et al. 2004 [34]	32; LVEF 22 ± 5%; NYHA II to IV; stable, optimised therapy	RCT versus sensory-threshold sham	Knee extensors and hamstrings, both legs	15 Hz	0.5 ms	25% to 30% of MVC	2 s on, 4 s off	4 h/day, 7 days/week, 10 weeks	Peak VO2 9.6 to 11.6 mL/kg/min; citrate synthase up, GAPDH down, MHC I up in biopsies
Deley et al. 2005 [50]	24 (12/12); LVEF NR; NYHA II to III; stable	RCT versus aerobic training	NR (knee extensor strength assessed)	Low frequency (Hz NR)	NR	NR	NR	5 h/week, 5 weeks	Peak VO2 and ventilatory threshold improved in both groups
Dobšák et al. 2006 [51]	30 (15/15); LVEF NR; NYHA II to III; home-based	RCT versus bicycle training	Quadriceps and calf, both legs	Low frequency (Hz NR)	NR	NR	NR	1 h/day, 7 days/week, 8 weeks	Alternative to aerobic training in functional endpoints
Karavidas et al. 2006 [52]	24 (16/8); LVEF <40%; NYHA II to III; stable	RCT (2:1) versus control	Lower limbs (detail NR)	NR	NR	NR	NR	6 weeks	FMD +38.5%; 6MWT +7.5%; TNF-alpha and sICAM-1 reduced
Banerjee et al. 2009 [53]	10; LVEF NR; stable chronic HF	Randomised crossover versus habitual activity	Major leg muscles	NR	NR	NR	NR	At least 1 h/day, 5 days/week, 8 weeks	Peak VO2 19.5 to 21.2 mL/kg/min; 6MWT 415 to 455 m; quadriceps strength up
Karavidas et al. 2013 [54]	30; HFpEF, LVEF 63 ± 6%; NYHA II to III	RCT versus placebo stimulation	Peripheral muscles (detail NR)	NR	NR	NR	NR	6 weeks	6MWT, quality of life, depressive symptoms and FMD improved; BNP not significantly changed
Labrunée et al. 2013, EMSICA [35]	11 NMES (LVEF 30 ± 3.6%) and 11 TENS (LVEF 24 ± 2.8%); NYHA III; stable	Randomised, double-blind, sham-controlled; acute	Quadriceps and triceps surae	25 Hz (NMES); 80 Hz (TENS)	200 µs	Maximal tolerated contraction (NMES); sensory only (TENS)	3 s on, 3 s off	Single 5 min session	MSNA 56.7 to 51.6 bursts/min after NMES; 69.7 to 63.5 after TENS; no change in BP or HR
Parissis et al. 2015 [55]	30; systolic HF, age 75 ± 3 years; NYHA II/III; stable	RCT versus placebo stimulation	Lower limbs (detail NR)	NR	NR	NR	NR	6 weeks	Quality of life, emotional status and FMD improved
Groehs et al. 2016 [36]	30 (15/15); LVEF ≤ 30%; NYHA IV; hospitalised, decompensated	RCT versus stimulation <20 mA	Lower limbs	10 Hz	150 ms (as printed)	70 mA	NR	60 min/day, 8 to 10 consecutive days	MSNA reduced; muscle blood flow, 6MWT, strength and quality of life improved
Kadoglou et al. 2017 [56]	120 (60/60); LVEF 28 ± 5%; NYHA II/III; age 71 ± 8 years; stable	RCT versus placebo stimulation; median follow-up 383 days	Lower limbs (detail NR)	NR	NR	NR	NR	6 weeks	HF hospitalisation HR 0.40 (95% CI 0.21 to 0.78); mortality unchanged
Iliou et al. 2017, CREMS-HF [57]	91 (50/41); LVEF 30 ± 7%; NYHA II/III; rehabilitation	RCT: exercise training plus EMS versus training alone	Quadriceps	10 Hz	NR	NR	NR	20 sessions	Peak VO2 +14% with EMS versus +15% without; no incremental benefit
Forestieri et al. 2018 [58]	70 randomised (35/35); advanced HF on intravenous inotropes; hospitalised	RCT versus usual care	Both lower extremities	NR	NR	NR	NR	Daily for about 2 weeks	Exercise tolerance (6MWT) improved with stimulation
Kondo et al. 2019 [10]	73 analysed (34/39); LVEF 37.5 ± 15.3% versus 42.9 ± 20.3%; NYHA III/IV; acute HF, hospitalised	RCT versus visible-contraction control	Quadriceps and triceps surae	200 Hz bursts followed by 20 Hz	NR	10% to 20% of MVC	NR	5 days/week from day after admission	Prespecified haemodynamic feasibility criteria met
Arenja et al. 2021 [59]	27 randomised (10/9/8); age > 60 years; acute HF; hospital then home	RCT: high- versus low-intensity EMS versus control	NR	NR	NR	High versus low intensity	NR	3 and 6 weeks	13 of 27 completed; intolerance of stimulation the main reason for dropout
Poltavskaya et al. 2022 [60]	45 (22/23); LVEF ≤ 40%, mean 32.3% and 30.8%; NYHA III/IV; hospitalised, decompensated	RCT versus sham stimulation	Anterior and posterior thigh and tibial muscles, both legs	25 Hz	1 ms	Visible contraction, then maximum tolerable	1 s on, 2 s off	30 to 90 min, 5 days/week, 7 to 10 sessions	Functional capacity and quality of life assessed at discharge and 1 month
Tanaka et al. 2022, ACTIVE-EMS [8]	31 (15/16); LVEF 43.4 ± 17.4%; NYHA III/IV in >80%; age ≥ 75 years; frail; acute HF, hospitalised	RCT: early rehabilitation plus EMS versus early rehabilitation	Quadriceps, hamstrings, tibialis anterior, triceps surae (belt electrodes)	20 Hz	250 µs	Maximum tolerable without pain	5 s on, 2 s off	30 to 40 min/day, 5 days/week, up to 2 weeks	Quadriceps strength and SPPB improved more with EMS; no adverse events
Ono et al. 2025 [9]	8; frail older adults with chronic HF; LVEF NR; home-based	Randomised crossover: rehabilitation plus NMES versus rehabilitation	Legs	50 Hz	NR	NR	NR	50 min/day, 5 days/week, 8 weeks	SPPB +2.67 points and faster sit-to-stand with NMES
Betz et al. 2026 [11]	30 (15/15); LVEF 26.8 ± 19.5% versus 30.0 ± 17.8%; severe HF or ≤6 months after transplantation; NYHA II/III; hospitalised; 60% with cardiac devices	RCT: physiotherapy plus NMES versus physiotherapy	Quadriceps, tibialis anterior, biceps femoris, gastrocnemius, both legs	2 to 12 Hz (week 1), 15 to 35 Hz (week 2)	300 µs	Maximum tolerable, 9 to 45 mA	Continuous at 2 to 5 Hz; 15 s on, 15 s off at ≥7 Hz	1 h/session, 2 weeks	StimFIT5 composite +8.2 versus +3.6 points; 6MWT +68 m; no device interference
Sendrowski et al. 2026 [61]	1; LVEF 20% to 25%; NYHA III; inpatient rehabilitation	Case report (WB-EMS added to rehabilitation)	Whole body (electrode suit)	85 Hz	350 µs	Tolerance (NR as % MVC)	4 s on, 4 s off	20 min, 3 sessions/week, 10 sessions	Serial biomarkers within safety limits; functional gains not attributable to WB-EMS alone

**Table 3:** Mechanistic claims made for skeletal muscle electrical stimulation in HFrEF, classified by the population in which each was demonstrated. Tier 1, demonstrated in patients with HFrEF; Tier 2, demonstrated in other human populations; Tier 3, demonstrated in animal or in vitro preparations; Untested, not measured after a programme of stimulation in any population. The tier describes the origin of the evidence, not its strength; the number of studies and their design are given for that purpose.

Claim	Tier	Evidence in HFrEF (Studies, Design, *n*, LVEF Criterion)	Evidence Elsewhere	What Would Move the Claim Up One Tier or Replicate It
Increases muscle strength	1	Repeated: uncontrolled [6,43], RCT versus training [49], crossover [53]; LVEF 20% to 30%; *n* = 7 to 46	Meta-analyses across HF [69,70]	Report evoked torque as % MVC to link dose to gain
Increases muscle volume	1	One uncontrolled study, *n* = 14, LVEF 22.3% [6]	Ageing muscle [62]; critical illness [78,79]	Imaging in a sham-controlled HFrEF trial
Improves peak VO2 and walking distance	1	Multiple RCTs, LVEF ≤ 40% [34,50,57]; meta-analyses [67,68,70]	Acute and frail HF with mixed LVEF [8,12]	Trials in contemporary HFrEF on foundational therapy with modern comparators
Low-frequency protocols do not raise cardiac output	1 (protocol-specific)	Direct measurement, *n* = 14 [6]	Contrast: shivering-type stimulation raises VO2 in healthy adults [65]	Haemodynamic monitoring across the frequency range in HFrEF
Improves endothelial function and lowers inflammatory markers	1	One RCT, *n* = 24, LVEF < 40% [52]	HFpEF [54]; older patients [55]	Replication with mechanistic separation of local and systemic effects
Acutely reduces muscle sympathetic nerve activity	1 (acute, short-term)	Two studies: sham-controlled acute, *n* = 11, LVEF 30% [35]; 8 to 10 days, *n* = 30, LVEF ≤ 30% [36]	Sensory stimulation (TENS) produces a similar acute effect [35]	MSNA after a multi-week programme
Shifts oxidative enzyme activity and MHC isoforms towards a slow phenotype	1 (single study)	One sham-controlled RCT with biopsy, *n* = 32, LVEF 22% [34]	Healthy volunteers [71]; heterogeneous non-HF review [72]	Replication with contemporary molecular panel [25]
Improves in vivo muscle energetics	Untested in effect (non-demonstration)	31P-MRS unchanged, *n* = 14, 5 weeks [6]	None	Adequately powered 31P-MRS or NIRS study
Reduces myofibrillar proteolysis	2	None	Cardiac surgery [80]; critical illness [78,79]; negative for muscle thickness [81]	Ubiquitin–proteasome markers in stimulated HFrEF biopsies
Activates mTOR or AMPK signalling; induces mitochondrial biogenesis	3	None	Isolated rat muscle [42]; reviews of ageing muscle [64]	Signalling assays in human HFrEF muscle after stimulation
Activates satellite cells and regeneration	2	None	Healthy older adults [62,63]	Satellite cell counts in HFrEF biopsies
Stimulation frequency determines fibre-type selectivity	3/not supported	None	Critical appraisal finds recruitment non-selective [38,39]	Restate as an inference; test with fibre typing
Attenuates ergoreflex (metaboreflex, mechanoreflex) sensitivity	Untested	None; prediction of the muscle hypothesis [26,27,30]	Exercise training alters reflex control and receptor expression [76]	Post-exercise circulatory occlusion before and after a stimulation programme
Reduces hospitalisation	1 (single study)	One placebo-controlled RCT, *n* = 120, LVEF 28% [56]	None	Multicentre outcome trial
Changes natriuretic peptides	Insufficient	Rarely measured; not significant where measured [13,54]	None	Systematic measurement in future trials

## Data Availability

No new data were created or analysed in this study. Data sharing is not applicable to this article.

## Abbreviations

The following abbreviations are used in this manuscript:

6MWT	six-minute walk test
31P-MRS	phosphorus-31 magnetic resonance spectroscopy
AMPK	AMP-activated protein kinase
BNP	B-type natriuretic peptide
BP	blood pressure
CIED	cardiac implantable electronic device
ESC	European Society of Cardiology
FES	functional electrical stimulation
FMD	flow-mediated dilatation
GAPDH	glyceraldehyde-3-phosphate dehydrogenase
HF	heart failure
HFpEF	heart failure with preserved ejection fraction
HFrEF	heart failure with reduced ejection fraction
HR	heart rate
LVEF	left ventricular ejection fraction
MHC	myosin heavy chain
MSNA	muscle sympathetic nerve activity
mTOR	mechanistic target of rapamycin
MVC	maximal voluntary contraction
NMES	neuromuscular electrical stimulation
NR	not reported
NYHA	New York Heart Association
PGC-1alpha	peroxisome proliferator-activated receptor gamma coactivator 1-alpha
PKB	protein kinase B
RCT	randomised controlled trial
sICAM-1	soluble intercellular adhesion molecule 1
SPPB	Short Physical Performance Battery
TENS	transcutaneous electrical nerve stimulation
TNF	tumour necrosis factor
TSC2	tuberous sclerosis complex 2
VO2	oxygen uptake
WB-EMS	whole-body electromyostimulation
